# Coding-complete genome sequence of an infectious laryngotracheitis virus from tracheal mucosa sample shipped on FTA card

**DOI:** 10.1128/mra.01390-25

**Published:** 2026-06-15

**Authors:** Renata Varga-Kugler, Zalan G. Homonnay, Istvan Kiss

**Affiliations:** 1 Ceva-Phylaxia Ltd. 87289 , Budapest, Hungary; Katholieke Universiteit Leuven, Leuven, Belgium

**Keywords:** infectious laryngotracheitis virus, Illumina, virus genome sequencing

## Abstract

Coding-complete sequence of a wild-type infectious laryngotracheitis virus was determined using Illumina iSeq 100 sequencing platform from broiler chicken tracheal mucosa samples transported on Flinders Technology Associates (FTA) cards, which are typically unsuitable for long read sequencing due to nucleic acid fragmentation.

## ANNOUNCEMENT

Infectious laryngotracheitis virus (ILTV) also known as *Iltovirus gallidalpha1*/gallid alphaherpesvirus 1 belonging to the family *Orthoherpesviridae* causes a highly contagious, economically important disease characterized by respiratory symptoms and egg production loss primarily in chickens (1). ILTV possesses a linear 150–155 kb long double stranded DNA genome encoding 80 open reading frames (ORFs) consisting of the unique long (UL) and unique short sequences flanked by inverted and terminal repeats (IR and TR) (2). Discrimination of vaccine and field ILTV strains from clinical samples is crucial for understanding epidemiology, outbreaks, and vaccine efficacy (1).

Whatman FTA cards stabilize and protect DNA at room temperature, so they are generally used for sample and nucleic acid transportation without special conditions (3). It is simple, but has several limitations, such as the fragmentation of nucleic acids (4, 5).

Tracheal mucosa samples collected in November 2024 from layer chickens were shipped to our laboratory on FTA cards from the Middle East. Since the samples tested positive for ILTV by qPCR (6), genome sequencing was performed to characterize the virus. Nucleic acid was extracted using QIAamp DNA Mini kit (QIAGEN): approximately 1 cm^2^ of FTA card was lysed in 300 µL buffer ATL for 10 min at room temperature. One hundred eighty microliters of this mixture was incubated at 56°C for 1 h with 20 µL Proteinase K and used in subsequent steps of “Protocol: DNA Purification from Dried Blood Spots” recommended by the manufacturer.

DNA library was prepared using Nextera XT DNA Library Preparation Kit and Nextera XT Index Kit v2 SetA (Illumina) from the DNA sample diluted to 0.2 ng/μL. Tagmentation and indexing reactions were reduced to half volume. PCR library was purified with Monarch Spin PCR&DNA Cleanup Kit (NEB) and diluted to 1 nM. Sequencing was performed on iSeq 100 sequencer using iSeq 100 i1 Reagent v2 (300-cycle) cartridge and flowcell (Illumina). Demultiplexing, adapter and index trimming were applied as implemented in the Illumina Local Run Manager “generate FASTQ” option.

**Fig 1:**
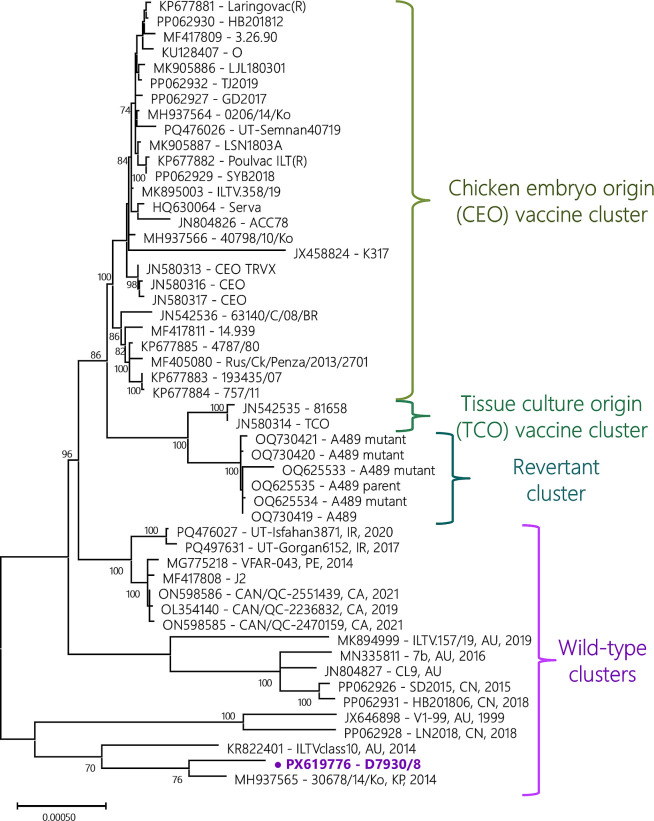
Maximum likelihood tree of ILTV strains based on whole genome sequences. Strain analyzed in the present paper is marked with purple dot. Clusters were defined, and reference sequences were selected according to Elshafiee et al. (7). Whole genome-based alignments were generated using the MAFFT algorithm implemented in Geneious Prime 2024.0.7 software. Phylogenetic tree was generated by MegaX software (8) using Tamura-3-parameter substitution model. Best-fit substitution model was selected based on the Bayesian information criterion as implemented in MegaX. Topology was validated by bootstrap analysis (100).

Paired-end sequencing resulted 10,471,582 reads (150-bases-long) of which 96,605 were mapped to ILTV sequence with accession number JN804826 as a reference using the Geneious mapper with medium sensitivity and trimming of the reads (10 bases) within the Geneious Prime 2024.0.7 software. Reference mapping obtained a 153,617 bp long consensus (width: 99.99%; mean coverage: 72.5×). Consensus was annotated based on sequence homology with reference and ambiguities were corrected manually within Geneious Prime. Assembly and editing resulted in a 152,684 bp long final consensus sequence with 48.1% G + C. Whole genome apart from an approximately 50 bp-long sequence in UL and approximately 16 bp-long in IR/TR region was determined. BLASTn v2.16.0 search found KR822401 as the most similar (99.75%) sequence in GenBank of the complete sequence and phylogenetic analysis revealed close relationship with wild-type ILTV strains from Australia and the Far East (Fig. 1).

The presented workflow proved to be suitable for determining the complete coding region of the ILTV genome from samples on FTA cards, enabling differentiation between vaccine and field strains. However, its success strongly depends on the quality of sampling and sample handling.

## Data Availability

This Whole Genome Shotgun project has been deposited in GenBank under accession no. PRJNA1367478. The coding-complete sequence of the ILTV D7930/8 was deposited in GenBank under accession number PX619776.
